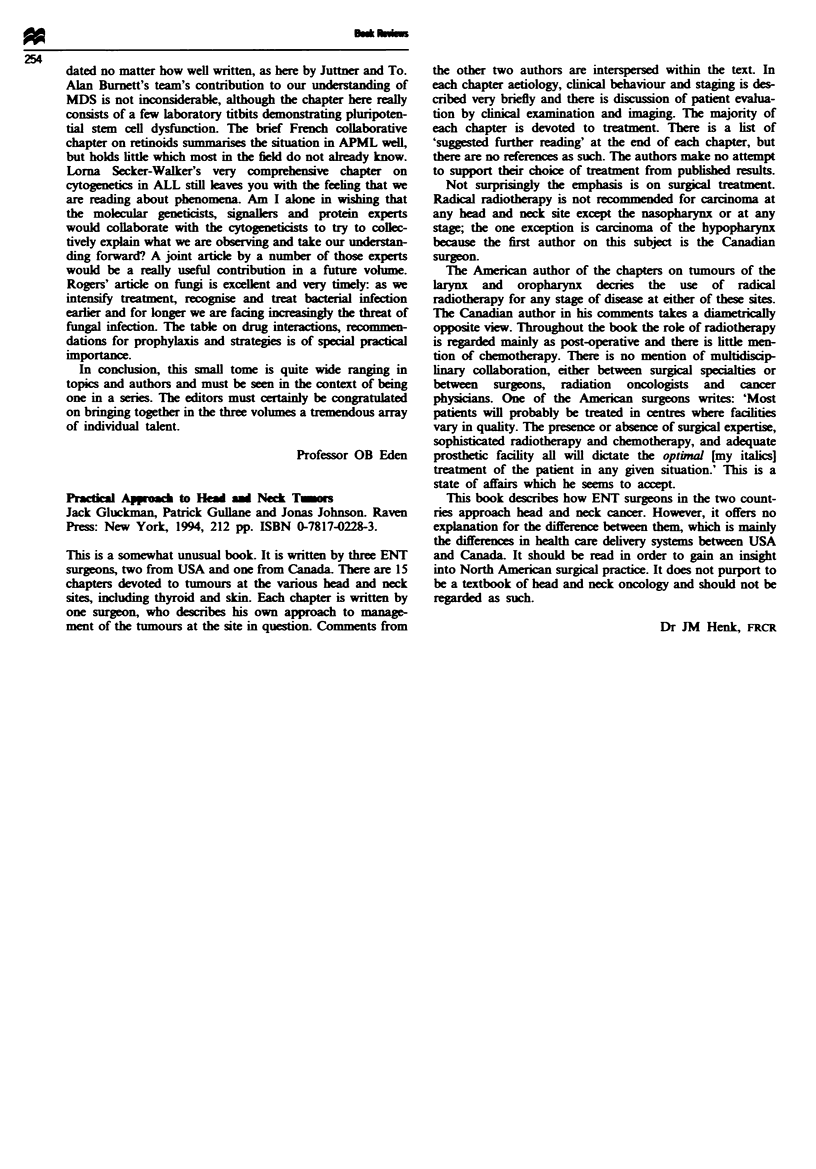# Practical Approach to Head and Neck Tumors

**Published:** 1995-07

**Authors:** JM Henk


**Practical Approach to Head and Neck Tumors**
Jack Gluckman, Patrick Gullane and Jonas Johnson. Raven Press: New York, 1994, 212 pp. ISBN 0-7817-0228-3.

This is a somewhat unusual book. It is written by three ENT surgeons, two from USA and one from Canada. There are 15 chapters devoted to tumours at the various head and neck sites, including thyroid and skin. Each chapter is written by one surgeon, who describes his own approach to management of the tumours at the site in question. Comments from the other two authors are interspersed within the text. In each chapter aetiology, clinical behaviour and staging is described very briefly and there is discussion of patient evaluation by clinical examination and imaging. The majority of each chapter is devoted to treatment. There is a list of 'suggested further reading' at the end of each chapter, but there are no references as such. The authors make no attempt to support their choice of treatment from published results.

Not surprisingly the emphasis is on surgical treatment. Radical radiotherapy is not recommended for carcinoma at any head and neck site except the nasopharynx or at any stage; the one exception is carcinoma of the hypopharynx because the first author on this subject is the Canadian surgeon.

The American author of the chapters on tumours of the larynx and oropharynx decries the use of radical radiotherapy for any stage of disease at either of these sites. The Canadian author in his comments takes a diametrically opposite view. Throughout the book the role of radiotherapy is regarded mainly as post-operative and there is little mention of chemotherapy. There is no mention of multidisciplinary collaboration, either between surgical specialties or between surgeons, radiation oncologists and cancer physicians. One of the American surgeons writes: 'Most patients will probably be treated in centres where facilities vary in quality. The presence or absence of surgical expertise, sophisticated radiotherapy and chemotherapy, and adequate prosthetic facility all will dictate the *optimal* [my italics] treatment of the patient in any given situation.' This is a state of affairs which he seems to accept.

This book describes how ENT surgeons in the two countries approach head and neck cancer. However, it offers no explanation for the difference between them, which is mainly the differences in health care delivery systems between USA and Canada. It should be read in order to gain an insight into North American surgical practice. It does not purport to be a textbook of head and neck oncology and should not be regarded as such.

Dr JM Henk, FRCR